# Supplementation of vitamin C promotes early germ cell specification from human embryonic stem cells

**DOI:** 10.1186/s13287-019-1427-2

**Published:** 2019-11-15

**Authors:** Zili Li, Fang Fang, Qian Zhao, Honggang Li, Chengliang Xiong

**Affiliations:** 1 Institute of Reproductive Health, Tongji Medical College, Huazhong University of Science and Technology, 13 Hangkong Road, Wuhan, 430030 China; 2 Wuhan Tongji Reproductive Medicine Hospital, 128 Sanyang Road, Wuhan, 430013 China; 3 Department of Obstetrics and Gynecology, Union Hospital, Tongji Medical College, Huazhong University of Science and Technology, Wuhan, 430022 China

**Keywords:** Embryonic stem cells, Primordial germ cells, Vitamin C, Epigenetic

## Abstract

**Background:**

As the precursors of sperm and eggs, human primordial germ cells (hPGCs) emerge as early as weeks 2 to 3 of post-implantation development. Recently, robust hPGC induction models have been established in vitro with different protocols, but global 5mC/5hmC epigenetic reprogramming is not initiated in vitro. Previous studies found that vitamin C can enhance Tet (ten-eleven translocation) enzyme expression and improve 5hmC level in cells. But the effect of vitamin C supplementation on hPGC in vitro induction is still unknown.

**Methods:**

We generated a gene-edited human embryonic stem cell (hESC) line carrying a BLIMP1-mkate2 reporter by CRISPR/Cas9 technology and used flow cytometry to optimize the PGC differentiation protocol; meanwhile, the expression of PGC genes (BLIMP1, TFAP2C, SOX17, OCT4) was evaluated by qRT-PCR. When different concentrations of vitamin C were added to the induction medium, the percentage of hPGCLCs (hPGC-like cells) was analyzed by flow cytometry; dot blot and ELISA were used to detect the levels of 5hmC and 5mC. The expression of TET enzymes was also evaluated by qRT-PCR.

**Results:**

We optimized the PGC differentiation protocol with the BLIMP1-mkate reporter hESCs, and the efficiency of PGC induction in vitro can be improved to 30~40%. When 50 μg/mL vitamin C was added, the derived hPGCLCs not only upregulated the expression of key genes involved in human early germ cell development such as NANOS3, TFAP2C, BLIMP1, and SOX17, but also increased the levels of 5hmC and TET enzymes.

**Conclusions:**

Taken together, supplementation of vitamin C can promote the in vitro induction of hPGCLCs from hESCs, which might be related to vitamin C-mediated epigenetic regulations during the differentiation process.

## Introduction

The germ cell lineage originates from primordial germ cells (PGCs) and serves as the foundation for heredity and evolution. In mammals, the mechanism for germ cell development has been extensively studied in the mouse models and significant progress has been made recently [1]. It has been reported that mouse PGC-like cells (mPGCLCs) can be induced from mouse embryonic stem cells (ESCs) and induced pluripotent stem cells (iPSCs), and the mPGCLCs were further differentiated into both sperm and egg, which could contribute to healthy offspring [1–4]. In contrast, due to the inaccessibility of human embryos in vivo and ethical limitations, the mechanisms underlying human germ cell development are still unclear. Moreover, significant differences exist in the mechanisms for germ cell development between human and mice [5, 6]. Thus, it is necessary to develop a proper in vitro research model for the precise understanding of human germ cell development.

Researchers have been working on the reconstitution of human germ cell development in vitro using human ESCs and iPSCs. Recently, human embryonic stem cells (hESCs) and human induced pluripotent stem cells (hiPSCs) have been induced into human primordial germ cell-like cells (hPGCLCs) in response to signals simulating the natural developing environment of hPGC in vivo, and several key questions during hPGC specification have been illuminated using this in vitro system. It has been demonstrated that SOX17 is a critical regulator of hPGC fate and that WNT signals induce the expression of EOMES to activate SOX17, which works together with TFAP2C to instate the hPGCLC transcriptional program, including the BLIMP1 expression [5, 7–9].

Notably, recent studies have investigated the genome-wide DNA demethylation dynamics in hPGCs. Consistent with the findings in mice, hPGCs undergo epigenetic reprogramming during early embryo development [10–13]. After colonization at embryonic gonads, the early hPGCs exhibit substantial demethylation (genome-wide 5-methylcytosine (5mC) level, ~ 20%), followed by further demethylation thereafter (genome-wide 5mC level, ~ 5%) [10, 11]. Thus, hPGCs present much lower genome-wide 5mC levels than inner cell mass cells of the blastocysts [10]. In addition, hPGCs display low levels of histone H3 lysine 9 di-methylation (H3K9me2) and histone H3 lysine 9 tri-methylation (H3K9me3) as well as of DNMT3A/3B and UHRF1, also suggesting epigenetic reprogramming at this stage [13–17].

The global DNA demethylation in the early germ line may be mediated by Tet enzymes, which convert 5mC to 5-hydroxy-methylcytosine (5hmC) [18, 19]. Ascorbate, the dominant form of vitamin C (Vc) under physiological pH conditions, is considered as an important cofactor for Tet enzymes and could regulate the epigenomic processes in mammalian cells [20–23]. Studies have shown that ascorbate causes the widespread DNA demethylation of many gene promoters and increases the 5hmC levels in many types of mammalian cells, including embryonic stem cells and mouse embryonic fibroblasts [24, 25]. Moreover, ascorbate also enhances the cell reprogramming process, which is usually accompanied by global DNA demethylation [21, 22]. Since hPGCs undergo complicated epigenetic reprogramming during early embryo development, Vc may play an important role in the hPGC differentiation process, which has not been reported currently. Therefore, the aim of the present work is to explore the effect of Vc supplementation on hPGC induction from hESCs in vitro.

## Materials and methods

### Culture of hESCs

The H1 hESCs were maintained under a feeder-free condition in TeSR-E8 medium (Stem Cell Technologies, 05990) on Matrigel (Corning, 356234)-coated cell culture plates. Cultures were passaged at a 1:10 to 1:20 split ratio every 4–6 days using 0.5 mM EDTA/PBS (Thermo Fisher, AM9260G). Ten micromolar Rho-associated protein kinase (ROCK) inhibitor Y-27632 (Selleck Chemicals, S1049) was added into the culture medium when passaging or thawing cells.

### Generation of BLIMP1-mkate knockin hESC lines

CRISPR gRNAs (Additional file 1: Tables) were designed to target the genomic sequences corresponding to the stop codon of BLIMP1 locus and cloned into px330 plasmid. The homology arms of donor construct were amplified by PCR using the primers in Additional file 1: Tables. The T2A-mkate2 fragment with hEF1a-Neo cassette flanked by loxP sites was amplified by PCR and inserted in frame at the 3-prime ends of the BLIMP1 coding sequences of the sub-cloned vector containing the homology arms by using Gibson Assembly Master Mix (NEB, E2611). The donor vectors (0.5 μg) and the Cas9 plasmids (0.5 μg) were transfected into the H1 ESCs (2 × 10^5^ cells per well of 6-well plate) by 3 μl of FuGENE 6 (Promega, E2691). After selection with 100 μg/ml G418 for 10 days, hESCs were dissociated into single cells and seeded at ~ 800 cells per 10-cm dish. Cells were allowed to grow until colonies from single cells became visible (~ 8–10 d). At this stage, single colonies were manually picked and seeded individually into 96-well plates. The targeted and random integrations were assessed by PCR on the extracted genomic DNA using the primer pairs listed in Additional file 1: Tables. Targeted alleles were further screened by Sanger sequencing to rule out indel mutations (Additional file 2: Figure S1C). The lines bearing the targeted insertion in BLIMP1 loci were transfected with a plasmid expressing Cre recombinase to remove the hEF1a-Neo-pA cassettes.

### Induction of hPGCLCs

For pre-induction, hESCs were dissociated with 0.5 mM EDTA/PBS, and 3 × 10^5^ cells per well were plated on Matrigel-coated 12-well plates in GK15 medium (G-MEM [Thermo Fisher, 11710-035], 15% KSR [Thermo Fisher, 10828-028], 0.1 mM NEAA [Thermo Fisher, 11140-050], 2 mM l-glutamine [Thermo Fisher, 35050-061], 1 mM sodium pyruvate [Thermo Fisher, 11360-070], 0.1 mM 2-mercaptoethanol [Sigma, M3148]) or aRB27 medium (Advanced RPMI 1640 [Thermo Fisher, 12633-012], 1% B-27 supplement [Thermo Fisher, 17504-044], 0.1 mM NEAA, 2 mM l-glutamine) with 3 μM CHIR (Selleck Chemicals, S2745), 40 ng/ml Activin A (PEPRO TECH, 120-14E), and 10 μM ROCK inhibitor. After 2 days of pre-induction, the cells were dissociated with Accutase (Thermo Fisher, A1110501) and plated into ultra-low cell attachment U-bottom 96-well plates (Corning, 7007) at a density of 2000–4000 cells per well to form embryoid bodies in 200 μl of PGC induction medium (GK15 medium or aRB27 medium containing 200~500 ng/ml BMP4 (R&D Systems, 314-BP-050), 10 ng/ml~1 μg/ml human LIF (R&D Systems, 7734-LF-100), 100 ng/ml SCF (R&D Systems, 255-SC-050), 50 ng/ml EGF (R&D Systems, 236-EG-200), and 10 μM ROCK inhibitor).

### Flow cytometry

The floating aggregates were dissociated with 0.05% Trypsin-EDTA/PBS for 15 min at 37 °C. After washing with PBS, the cell suspension was filtered by cell strainer to remove cell clumps and then subjected to centrifugation. To analyze hPGCLCs or hiPSCs with cell surface markers, the dissociated cells were stained with PE-conjugated anti-human CD326 (EpCAM), FITC-conjugated anti-human/mouse CD49f (INTEGRINα6), PE-conjugated anti-TRA-1-60, or FITC-conjugated anti-SSEA-4. Intracellular staining was performed using a BD kit (BD, 560589) according to the manufacturer’s instructions with PerCP-Cy™5.5 Mouse anti-Oct3/4, PE Mouse anti-human Nanog, or Alexa Fluor® 647 Mouse anti-Sox2. The primary antibodies used in this study are listed in Additional file 1: Tables. The stained cells were resuspended in PBS and analyzed with a flow cytometer (Beckman, DxFLEX).

### Quantitative RT-PCR

Total RNA was extracted using TRIzol regent (Thermo Fisher, AM9738), and cDNA was synthesized using Reverse Transcription Kit (Takara, RR047A). The qRT-PCR was performed using SYBR Premix Ex Taq II (Takara). The primers used are shown in Additional file 1: Tables. Values normalized to GAPDH and relative to control samples are shown. Error bars are mean ± SD from three independent experiments.

### Dot blot

Dot blot assay was performed according to published protocols [26].

### Quantification of 5hmC and 5mC

Quantification of 5hmC and 5mC were performed using MethylFlash™ Global DNA Hydroxymethylation (5-hmC) ELISA Easy Kit (A-P-1032, Colorimetric) and MethylFlash™ Global DNA Methylation (5-mC) ELISA Easy Kit (A-P-1030, Colorimetric) according to the manufacturer’s instructions.

## Results

### Establishment of hESCs bearing BLIMP1-mkate2 reporter

**Fig. 1:**
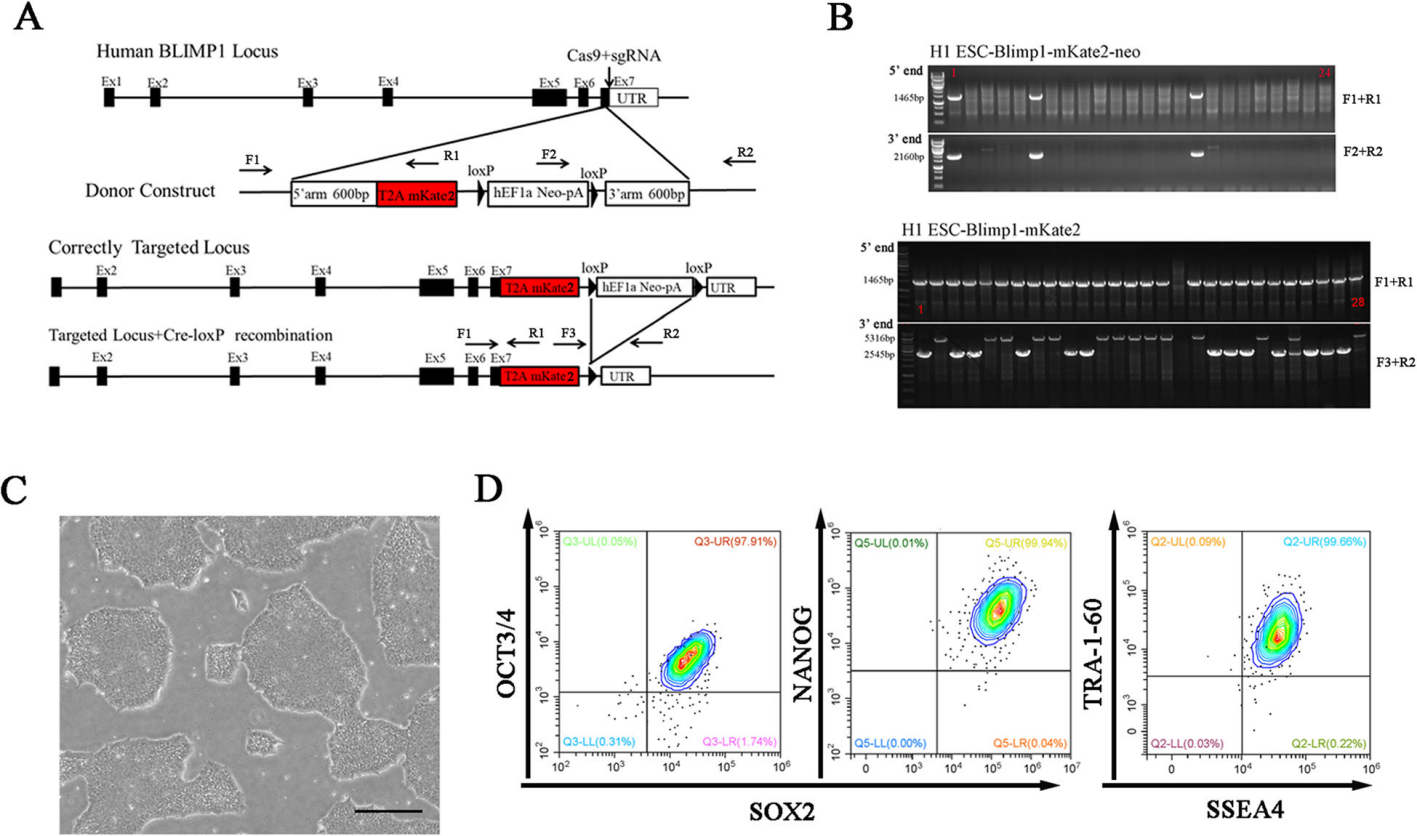
Generation of BLIMP1-mkate2 reporter knockin hESC lines. **a** Schematic illustration of the BLIMP1 locus, and the donor construct carrying T2A-mKate2 and hEF1a-Neo-pA fragments. Black boxes indicate the exons. **b** Screening by PCR of the homologous recombinants for BLIMP1-mkate2 and of the removal of the selection cassettes (loxP-hEF1a-Neo-pA-loxP). The clones bearing BLIMP1-mkate2 were selected for use in the subsequent studies. **c** A phase-contrast image of the BLIMP1-mkate2 reporter knockin hESCs. Scale bar = 200 μm. **d** FACS analysis for OCT3/4, SOX2, SSEA4, TRA-1-60, and NANOG expression in reporter knockin hESCs

BLIMP1 has been reported to be expressed in hPGCs and human fetal germ cells [12]. The expression of BLIMP1 in human early germ cells represses the somatic programs and activates and stabilizes the germline transcriptional circuit [7, 8]. First, we generated a BLIMP1-mkate2 knock-in reporter in H1 hESCs by CRISPR/Cas9 strategy as described in Fig. 1a. The genome-edited ESCs were maintained under a feeder-free, defined condition with basic fibroblast growth factor (bFGF) on the Matrigel. After selection with geneticin, we seeded the survived cells at single-cell level and picked out single colonies several days later for verification of homologous recombination by PCR (Fig. 1b, Additional file 2: Figure S1B), and we expanded the correctly targeted clones with normal karyotype (Additional file 2: Figure S1A) for following studies. Meanwhile, the derived BLIMP1-mkate2 reporter knock-in hESCs exhibited typical morphology of hESCs and expressed the pluripotent markers such as NANOG, OCT3/4, SOX2, SSEA4, and TRA-1-60 (Fig. 1c, d).

### Induction of hPGCLCs from hESCs bearing BLIMP1-mkate2 reporter

**Fig. 2:**
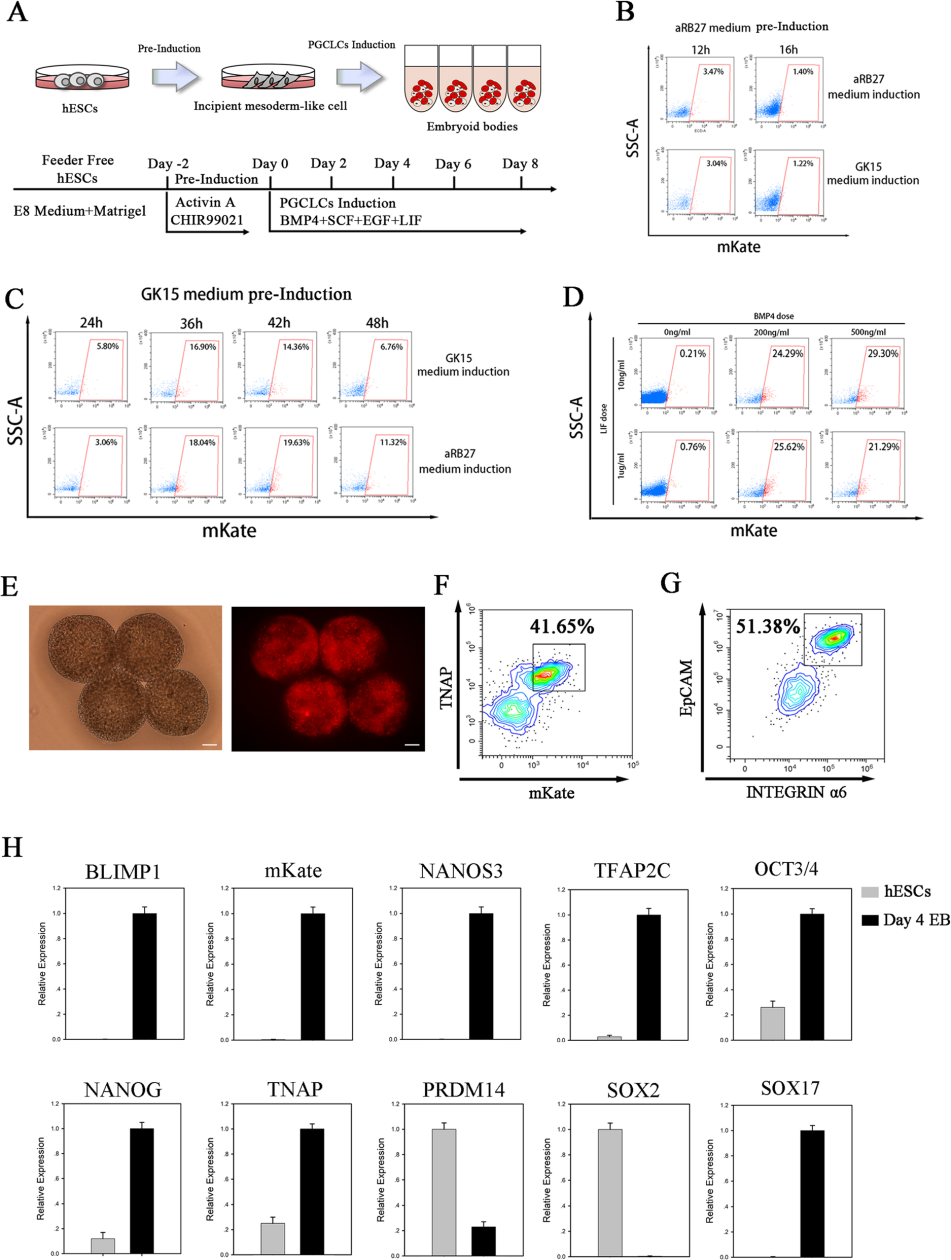
Induction of PGCLCs from BLIMP1-mkate2 reporter knockin hESCs with a two-step method. **a** Schematic protocol for PGCLCs induction. **b**, **c** FACS analysis of mKate (+) cell induction by aRB27 medium and GK15 medium at day 4 with different pre-induction time; *n* = 3 independent experiments. **d** FACS analysis of mKate (+) cell induction at day 4 stimulated by different concentrations of LIF (1 μg/ml or 10 ng/ml) and BMP4 (0, 200, 500 ng/ml); *n* = 3 independent experiments. **e** Bright field (left) and fluorescence images (right) of day 4 embryoid bodies (EBs) stimulated by cytokines; scale bar = 200 μm; *n* = 3 independent experiments. **f** FACS analysis of mKate and TNAP double-positive cells at day 4; *n* = 3 independent experiments. **g** FACS analysis of EpCAM and INTEGRINα6 double-positive cells at day 4; *n* = 3 independent experiments. **h** Expression analysis by RT-qPCR for day 4 EBs differentiated form BLIMP1-mkate2 reporter knockin hESCs. Relative expression levels are shown with normalization to hESCs or day 4 EB. *n* = 3 independent experiments. Data are presented as mean ± SD

In recent years, hPGCLCs have been induced from human pluripotent stem cells (hPSCs) in vitro through a two-step method using different basal media (aRB27 and GK15) containing different cytokines [8, 9] (Fig. 2a). Based on these published methods, we tried to optimize and develop a more efficient protocol for hPGC induction in vitro with the BLIMP1-mkate2 reporter cells. We tested different combinations of basal media and found that aRB27 medium used in pre-induction stage did not confer hESCs with competence for germline fate no matter what type of basal medium (GK15 or aRB27) was used in the following induction stage (0–4% BLIMP1-mkate2-positive putative hPGCLCs at day 4 of induction) (Fig. 2b). But when the cells were pre-induced in GK15 medium for 36–42 h and then induced in aRB27 medium, the intensity of BLIMP1-mkate2 reporter increased sharply, resulting in about 18–19% BLIMP1-mkate2-positive putative hPGCLCs at day 4 of induction (Fig. 2c). Moreover, we optimized the concentration of BMP4 and LIF, which were important cytokines involved in PGC differentiation. We observed that the efficiency for the induction of BLIMP1-mkate2-positive cells was the highest when we used 500 ng/ml of BMP4 and 10 ng/ml of LIF in the induction stage (Fig. 2d, e). The FACS analyses revealed that the average percentage of mkate2/TNAP double-positive cells per embryoid body at day 4 was around 41% (Fig. 2f). Consistently, about 50% of the cells became double-positive for EpCAM and INTEGRINα6 (Fig. 2g). We also examined the gene expression dynamics during the mkate2-positive hPGCLC induction by qPCR. At day 4 of the induction stage, the embryoid bodies upregulated key PGC genes (BLIMP1, NANOS3, TFAP2C, TNAP) and pluripotent genes (OCT4 and NANOG) (Fig. 2h). SOX17 has been reported to be involved in human early germ cell fate [7]. Notably, SOX17 was significantly upregulated, whereas SOX2 was repressed at day 4. Interestingly, expression of two mouse PGC regulators, PRDM14 and T, are not activated at this stage, and the day 4 cells also remained low or negative expression for DPPA3 and late PGC genes (DAZL and DDX4) (Additional file 2: Figure S1D).

### Vitamin C enhances the efficiency of hPGCLC induction in vitro

**Fig. 3:**
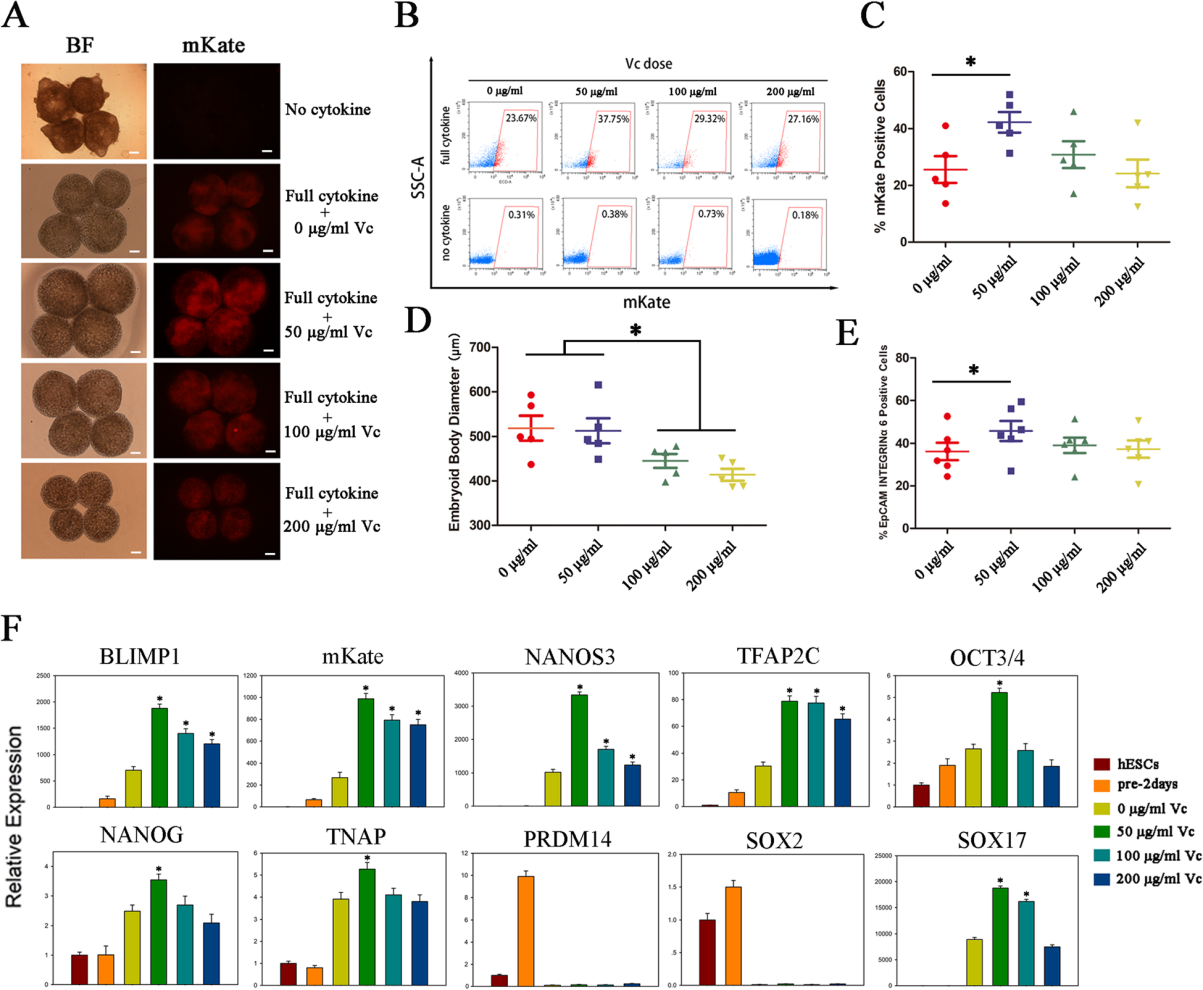
Effect of vitamin C supplementation on PGCLC induction. **a** Bright field and fluorescence images of day 4 EBs stimulated by no cytokines or by various concentrations of vitamin C (0, 50, 100, 200 μg/ml) in addition to cytokines; *n* = 5 independent experiments. **b** FACS analysis of mKate (+) cells induction stimulated by various concentrations vitamin C (0, 50, 100, 200 μg/ml) with or without cytokines; *n* = 5 independent experiments. **c** Scatter dot plot for the percentage of mKate (+) cells in day 4 EBs stimulated by different concentrations of vitamin C (0, 50, 100, 200 μg/ml); *n* = 5 independent experiments. Data are presented as mean ± SD. Statistical analysis was performed by one-way analysis of variance. *p < 0.05. **d** Scatter dot plot for diameters of day 4 EBs stimulated by different concentrations of vitamin C (0, 50, 100, 200 μg/ml); *n* = 5 independent experiments. Data are presented as mean ± SD. Statistical analysis was performed by one-way analysis of variance. *p < 0.05. **e** Scatter dot plot for the percentage of EpCAM and INTEGRINα6 double-positive cells in day 4 EBs stimulated by different concentrations of vitamin C (0, 50, 100, 200 μg/ml); *n* = 6 independent experiments. Data are presented as mean ± SD. Statistical analysis was performed by one-way analysis of variance. *p < 0.05. **f** Expression analysis by RT-qPCR for day 4 EBs stimulated by different of concentrations of vitamin C (0, 50, 100, 200 μg/ml). Relative expression levels are shown with normalization to hESCs. *n* = 3 independent experiments. Data are presented as mean ± SD. Statistical analysis was performed by one-way analysis of variance. *p < 0.05

To clarify the influence of vitamin C on hPGCLC induction in vitro, we added different concentrations of vitamin C to the induction media containing full cytokines and evaluated the percentage of mkate2-positive cells. The embryoid bodies maintained in media without cytokines did not show mkate2 expression after 4 days of induction, and the shape of them is irregular. With the stimulation of full cytokines and vitamin C, the percentage of mkate2-positive cells in day 4 embryoid bodies reached the highest in 50 μg/ml of vitamin C group (Fig. 3a–c). Meanwhile, the mean diameters of the embryoid bodies decreased with increasing vitamin C concentrations from 50 to 200 μg/mL, and the embryoid bodies were significantly smaller in the 200 μg/ml of vitamin C group than those in the other groups (Fig. 3d). Consistently, FACS analyses of both the reporter and native hESCs revealed that the average percentage of the EpCAM/INTEGRIN α6 double-positive cells at day 4 was the highest when we used 50 μg/ml of vitamin C in the induction medium (Fig. 3e, Additional file 3: Figure S2A, B). Moreover, we observed that the embryoid bodies exhibited the highest levels of the key PGC genes (NANOS3, BLIMP1, TFAP2C, TNAP), as well as pluripotent genes (OCT4, NANOG) at day 4 of stimulation with 50 μg/ml vitamin C (Fig. 3f). However, all the cells in different groups remained low or negative expression for DPPA3 and late PGC genes (DAZL and DDX4) and also modest level for T (Additional file 3: Figure S2C). All these results suggested that supplementation of Vc could enhance the efficiency of hPGCLC induction in vitro, but the derived cells were still at early stage.

### Vitamin C promotes the expression of TET enzymes and increases the 5hmc level in hPGCLCs

**Fig. 4:**
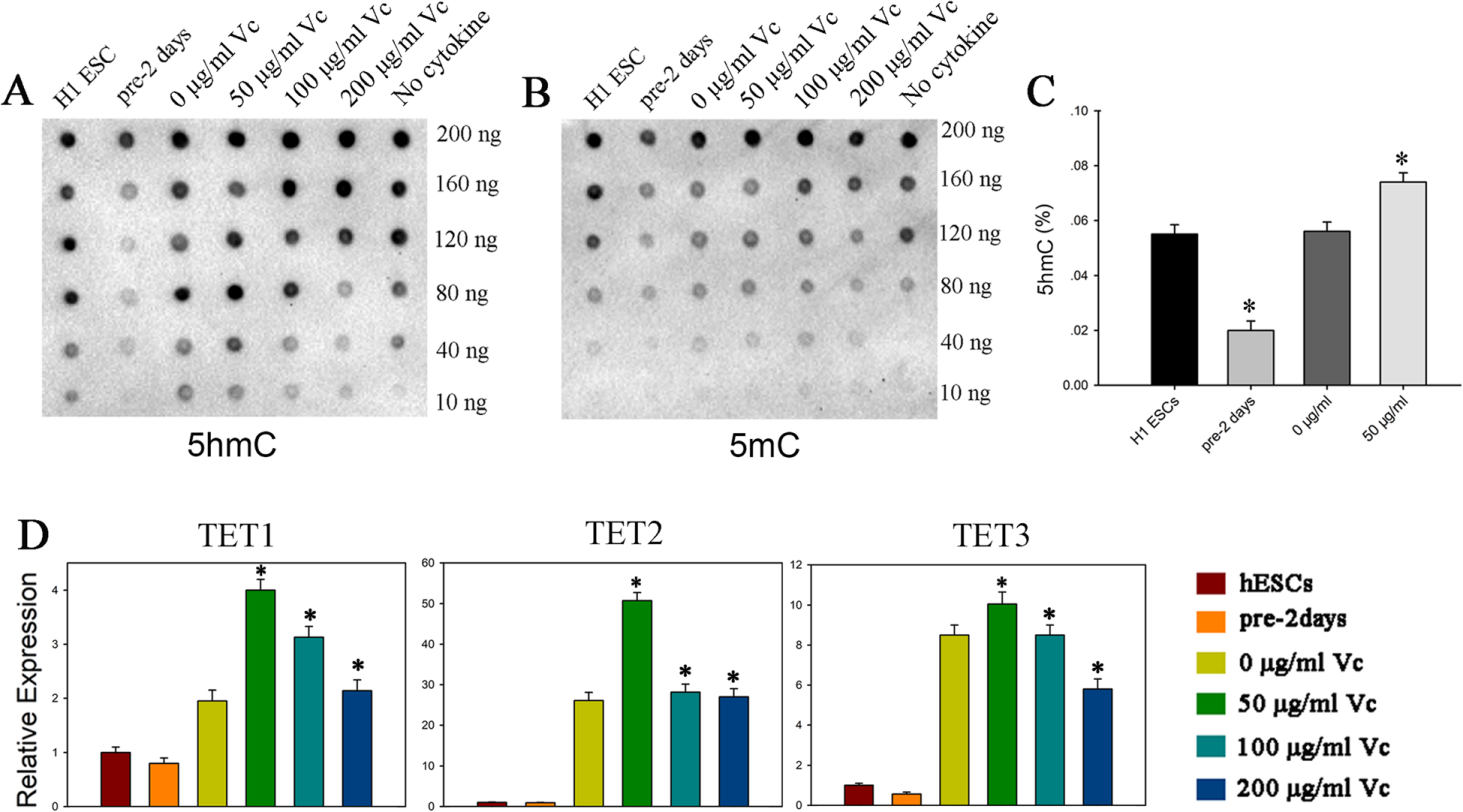
Epigenetic analyses of PGCLC induction stimulated by vitamin C. **a**, **b** Global 5hmC and 5mC levels detected by dot blot in different concentrations of vitamin C (0, 50, 100, 200 μg/ml); *n* = 3 independent experiments. **c** Analyses of 5hmC levels by ELISA; *n* = 3 independent experiments. Data are presented as mean ± SD. Statistical analysis was performed by one-way analysis of variance. *p < 0.05. **d** Expression analysis of epigenetic regulators by RT-qPCR for day 4 EBs stimulated by different of concentrations of vitamin C (0, 50, 100, 200 μg/ml). Relative expression levels are shown with normalization to hESCs; *n* = 3 independent experiments. Data are presented as mean ± SD. Statistical analysis was performed by one-way analysis of variance. *p < 0.05

We next used dot blot and ELISA Easy Kit to detect the 5mC and 5hmC levels during the differentiation process. The results revealed that compared with the other groups, the day 4 cells treated with 50 μg/ml of vitamin C exhibited global increase in 5hmC levels (Fig. 4a, c). In contrast, there is no significant difference in global 5mC levels among day 4 cells treated with different concentrations of vitamin C, even though the global 5mC levels of day 4 cells were all lower than those of ESCs, which indicated a global DNA demethylation in hPGCLCs (Fig. 4b and Additional file 4: Figure S3). In the mouse PGCs, loss of 5mC could be coupled with the conversion of 5mC to 5hmC and TET enzymes, especially for TET1 and TET2, mainly convert 5mC to 5hmC. So we also examined the expression of TET enzymes during hPGCLC induction. We found that the day 4 embryoid bodies upregulated TET enzymes (TET1, TET2, TET3), compared with hESCs and pre-induced cells. Moreover, the day 4 embryoid bodies treated with 50 μg/ml of vitamin C displayed the highest levels of TET genes, which indicated DNA demethylation in hPGCLC induction (Fig. 4d).

## Discussion

The generation of human germline cells in vitro requires reconstitution of the complicated pathways of germ cell development. Two previous groups reported the specification of hPGCLCs from hPSCs with different two-step methods, which have established a foundation for studies on the mechanism of hPGC specification [7, 8]. The hPSCs were first pre-induced adherently with different culture conditions to get into an intermediate state, and thereafter, thousands of pre-induced cells were cultured under a floating condition in the presence of similar signaling molecules (BMP2 or BMP4, SCF, EGF, and LIF) to form embryoid bodies. After 6–8 days, hPGCLCs positive for early hPGC markers were detected in the embryoid bodies, but these cells were still negative for late PGC genes (DAZL and DDX4) and evidently represent the earliest stages of the human germ cell lineage. Here, we set out to establish hESCs bearing BLIMP1-mkate2 reporter to monitor the process of hPGC specification and demonstrated that the reporter hESCs could be differentiated into early hPGCLCs in vitro*.* Moreover, with the BLIMP1-mkate2 reporter, we optimized the previous induction methods and developed a more efficient protocol for hPGC induction in our lab.

In mammals, global epigenetic reprogramming occurs during PGC development to erase parental epigenetic memories and facilitate germ cell differentiation [6, 27, 28]. In mice, PGCs undergo genome­wide DNA demethylation as they migrate and colonize the genital ridge from embryonic day 7.5 (E7.5) to E13.5 [12, 15, 29]. Similarly, hPGCs also exhibit overall DNA demethylation in week 8 embryos when they settle in the genital ridge. And the DNA methylation further dropped to the lowest level in the male PGCs of week 11 embryos, with only 7.8% methylation remaining in the whole genome [11]. Recent evidence suggests that the enzymatic conversion of 5mC to 5hmC plays an important role in DNA demethylation. TET enzymes (TET1, TET2, and TET3) oxidize 5mC to 5hmC, and further to 5-formylcytosine (5fC) and to 5-carboxylcytosine (5caC), which are ultimately replaced by unmodified cytosine, to mediate the DNA demethylation [18, 19, 30, 31]. Notably, hPGCs exhibit transiently high levels of 5hmC, which are coupled with TET1 and TET2 upregulation from week 4 to week 11 [11].

The TET family of DNA hydroxylases is included in the diverse group of alpha-ketoglutarate-dependent dioxygenases (α-KGDDs), which function as erasers of epigenetic modifications and are activated by ascorbate [23]. Interestingly, Chen et al. reported that TET1, in an ascorbate-dependent manner, regulated 5hmC formation at loci critical for the somatic cell reprogramming [22]. In the absence of all three TET proteins, TET TKO mouse embryonic fibroblasts fail to be reprogrammed because of a block in the mesenchymal-to-epithelial transition (MET) step [32]. Similar to its role in somatic cell reprogramming, vitamin C has been shown to maintain the proliferation and differentiation potential of stem cells, like ESCs, iPSCs, neural stem cells (NSCs), and mesenchymal stem cells (MSCs) [33]. For instance, vitamin C could enhance the differentiation of NSCs toward dopamine neurons through boosting of TET1 and JMJD3 activity [34]. In hematologic malignancies, vitamin C treatment might enhance the enzymatic activity of TET2 to promote 5hmC formation and DNA demethylation in myelodysplastic syndrome or acute myeloid leukemia cells [35]. Yin et al. reported that ascorbate directly enhanced the activity of purified C-terminal catalytic domain of TET2 to oxidize 5mC to 5hmC and 5fC [36]. Furthermore, 5hmC level was decreased in various tissues of l-gulonolactone oxidase (GULO) knockout mice, which lost the ability to synthesize ascorbate [23]. The accumulating evidence strongly indicates that ascorbate not only is a cofactor for TET dioxygenases in the conversion of 5mC to 5hmC, but also mediates TET induction, leading to DNA demethylation [36]. As a result, we speculate that varying levels of ascorbate might affect the epigenetic reprogramming of PGCs during embryonic development.

Unlike most other mammals, including many primates and mice, humans are incapable of synthesizing vitamin C due to a loss of function mutation in the GULO gene that is required to catalyze the final step of vitamin C formation in the liver [37]. However, studies on the effects of ascorbate supplementation on hPGC specification and DNA demethylation dynamics are still lacking to date. Therefore, we set out to test the effect of ascorbate (vitamin C) supplementation at different concentrations on the induction of hPGCLCs in vitro with the BLIMP1-mkate2 reporter hESCs. We found that addition of vitamin C at 50 μg/ml to hESCs improved the efficiency of hPGCLC induction most significantly, along with higher levels of TET enzymes and 5hmC in the derived hPGCLCs than other groups. However, there is no significant difference in global 5mC levels among day 4 cells treated with different concentrations of vitamin C. In contrast, Sosa et al. reported that the rhesus macaque PGCLCs (rPGCLCs) generated in aggregate differentiation in vitro corresponded to early rPGCs prior to global 5mC erasure, even though the rPGCLCs were all 5hmC positive [38]. Thus, our results reveal that supplementation of vitamin C might propel the global 5mC/5hmC epigenetic reprogramming during the differentiation process in vitro.

## Conclusions

In conclusion, we demonstrated that the supplementation of vitamin C can promote in vitro induction of hPGCLCs, accompanied by increased levels of 5hmC and TET enzymes during the differentiation process. Nevertheless, much work remains to be carried out to investigate the exact role of vitamin C during hPGCLC specification in vitro and its related epigenetic mechanism. Potential applications of vitamin C in the clinic, like reproductive medicine or cancer therapy, also deserve further exploration.

## Supplementary information

**Additional file 1:** **Tables.** Primers and antibodies used in this study.

**Additional file 2: Figure S1.** Properties of BLIMP1-mkate2 reporter knockin hESCs lines. (A) The reporter knockin hESCs lines bear a normal karyotype (46, XY). (B) The knockin hESCs lines bear the BLIMP1-mkate2 reporter in a homozygous fashion. (C) Sequencing results of knockin cell line. (D) Expression analysis of late PGC genes by RT-qPCR in day 4 EBs. Relative expression levels are shown with normalization to hESCs or day4 EB. *n*=3 independent experiments; Data are presented as mean ± SD. n.d., not detected.

**Additional file 3: Figure S2.** (A) FACS analysis of non-knockin H1 ESCs with EpCAM and INTEGRINα6 antibodies at day4; n=3 independent experiments; Data are presented as mean ± SD; Statistical analysis was performed by one-way analysis of variance. *p <0.05. (B) Expression analysis by RT-qPCR for day 4 EBs differentiated form native hESCs; Relative expression levels are shown with normalization to hESCs or day 4 EBs. n=3 independent experiments; Data are presented as mean ± SD. (C) Expression analysis of late PGC genes by RT-qPCR for day 4 EBs stimulated by different concentrations of Vitamin C (0, 50, 100, 200μg/ml). Relative expression levels are shown with normalization to hESCs. Error bars indicate mean ± SD from three independent biological replicates. n.d., not detected.

**Additional file 4: Figure S3.** Analysis of 5mC levels by ELISA. n=3 independent experiments; Data are presented as mean ± SD; Statistical analysis was performed by one-way analysis of variance. *p <0.05.

## Data Availability

All relevant data are available from the authors upon reasonable request.

## Abbreviations

5hmC: 5-Hydroxy-methylcytosine
5mC: 5-Methylcytosine
ESCs: Embryonic stem cells
iPSCs: Induced pluripotent stem cells
MET: Mesenchymal epithelial transformation
MSCs: Mesenchymal stem cells
NSCs: Neural stem cells
PGCs: Primordial germ cells
TET: Ten-eleven translocation
