# Supplementary material for: Supplementation of vitamin C promotes early germ cell specification from human embryonic stem cells
Source: Stem Cell Res Ther. 2019 Nov 15;10:324. doi: 10.1186/s13287-019-1427-2 (PMC6858754; doi:10.1186/s13287-019-1427-2)
Supplement: Supplementary file 1 — Additional file 1: Tables. Primers and antibodies used in this study. [file 13287_2019_1427_MOESM1_ESM.docx]

**Primers used in this study**

**Knockin primers and Genotyping primers**

| **Name** | **Forward primer 5’-3’** | **Reverse primer5’-3’** |
| --- | --- | --- |
| BLIMP1-5arm | GGTAAGCTTACGCGTGGTCTGCCACAAGAGATTTAGC | CCAGATCCAGGATCCATTGGTTCAACTGTTTC |
| BLIMP1-3arm | TATACGAAGTTATGATTTTCAGAAAACACTTATTTTGTTTCTTAAGTTATGAC | GCGAATTCGGTACCGGTCCAACATTGGCAATTACCC |
| T2A-mKate2-Ef1a-neo-PA | TGGATCCTGGATCTGGCGAGGGCCGC | TGAAAATCATAACTTCGTATAGCATACATTATACGAAGTTATAAGCTTCAGGTCGAGG |
| sgRNA | TTACAGGTACCAGAGGTAGT |  |
| F1+R1 | CAGCTTCCAGTGGGTGGGAAAATGGTA | TCCACATAGTAGACGCCGGGCATCTT |
| F2+R2 | CTGACCGCTTCCTCGTGCTTTACGGTA | TGGAGCACATTTACTCGGTTTCTCCTCTA |
| F3 | TACGGCAGCAAAACCTTCATCAACCACA |  |

**Q-PCR primers**

| **Name** | **Forward primer 5’-3’** | **Reverse primer5’-3’** |
| --- | --- | --- |
| GAPDH | CGCTTCGCTCTCTGCTCCTCCTGT | GGTGACCAGGCGCCCAATACGA |
| BLIMP1 | CGGGGAGAATGTGGACTGGGTAGAG | CTGGAGTTACACTTGGGGGCAGC |
| mKate2 | TGTCTAAGGGCGAAGAGCTG | ATTCTCATGGTCTGGGTGCC |
| NANOS3 | CCCGAAACTCGGCAGGCAAGA | AAGGCTCAGACTTCCCGGCAC |
| TFAP2C | CGCTCATGTGACTCTCCTGACATCC | TGGGCCGCCAATAGCATGTTCT |
| OCT3/4 | GCTGGAGCAAAACCCGGAGG | TCGGCCTGTGTATATCCCAGGGTG |
| NANOG | TGCTGAGATGCCTCACACGGA | TGACCGGGACCTTGTCTTCCTT |
| TNAP | AAGCAGGTCTTGGGGTGCACCA | TTGGTCTCGCCAGTACTTGGGGT |
| PRDM14 | CTACCGAGCCCGAGTGGCCTAC | TAGAGCCATCCCGGGACCGCA |
| SOX2 | ACACCAA TCCCA TCCACACT | CCTCCCCAGGTTTTCTCTGT |
| SOX17 | GAGCCAAGGGCGAGTCCCGTA | CCTTCCACGACTTGCCCAGCAT |
| TET1 | GGGCACCCTACCGACAGAAGATGC | CTTCTGGGGCTTGGGCTTCTACC |
| TET2 | GGATGTCCTATTGCTAAGTGG | GAATCACAATCACTGCAGCCTC |
| TET3 | GAGCTGGCGGGCATTACG | TGCGGCTCCACCTTGAGG |
| DNMT1 | GGAGAGGCTAAGCGTTCAAG | AAATGAGATGTGATGGTGGTTTG |
| DNMT3A | AAGAGCACAGCGGAGAAG | GCAGATGTCCTCAATGTTCC |
| DNMT3B | CCATGAAGGTTGGCGACAA | TGGCATCAATCATCACTGGATT |
| DPPA3 | AAGCCCAAAGTCAGTGAGATGA | GCTATAGCCCAACTACCTAATGC |
| DAZL | TGGCCCTTCTTTCAGTGACTTC | GACCCTAGGGGGCACTAGTAA |
| DDX4 | TTCTTCACAAGCTCCCAATCCA | TTCTTCTCTGCATCAAAACCACA |
| T | AGCCAAAGACAATCAGCAGAAA | CACAAAAGGAGGGGCTTCACTA |

**Antibodies Used in This Study**

| **Antibody** | **Company** | **Cat NO.** |
| --- | --- | --- |
| OCT3/4 | BD Biosciences | 560589 |
| NANOG | BD Biosciences | 560589 |
| SOX2 | BD Biosciences | 560589 |
| TRA-1-60 | BD Biosciences | 560884 |
| SSEA4 | BD Biosciences | 560308 |
| TNAP | BD Biosciences | 561495 |
| EpCAM | BioLegend | 324228 |
| INTEGRIN α6 | BioLegend | 313608 |
| 5hmc | abcam | ab106918 |
| 5mc | abcam | ab10805 |
