# Supplementary figures and images for: Supplementation of vitamin C promotes early germ cell specification from human embryonic stem cells
Source: Stem Cell Res Ther. 2019 Nov 15;10:324. doi: 10.1186/s13287-019-1427-2 (PMC6858754; doi:10.1186/s13287-019-1427-2)

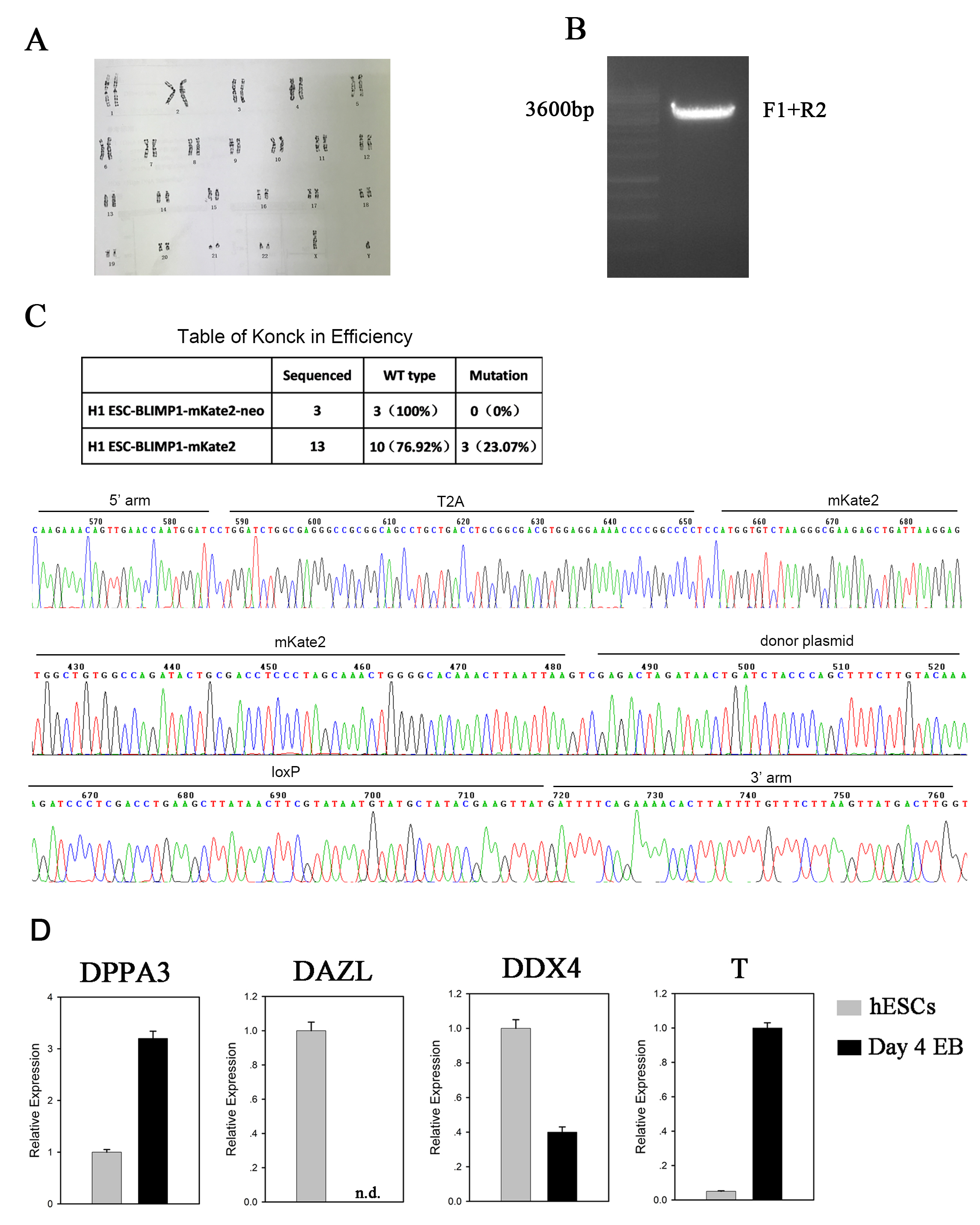

Supplement: Supplementary file 2 — Additional file 2: Figure S1. Properties of BLIMP1-mkate2 reporter knockin hESCs lines. (A) The reporter knockin hESCs lines bear a normal karyotype (46, XY). (B) The knockin hESCs lines bear the BLIMP1-mkate2 reporter in a homozygous fashion. (C) Sequencing results of knockin cell line. (D) Expression analysis of late PGC genes by RT-qPCR in day 4 EBs. Relative expression levels are shown with normalization to hESCs or day4 EB. n=3 independent experiments; Data are presented as mean ± SD. n.d., not detected. [file 13287_2019_1427_MOESM2_ESM.tif]

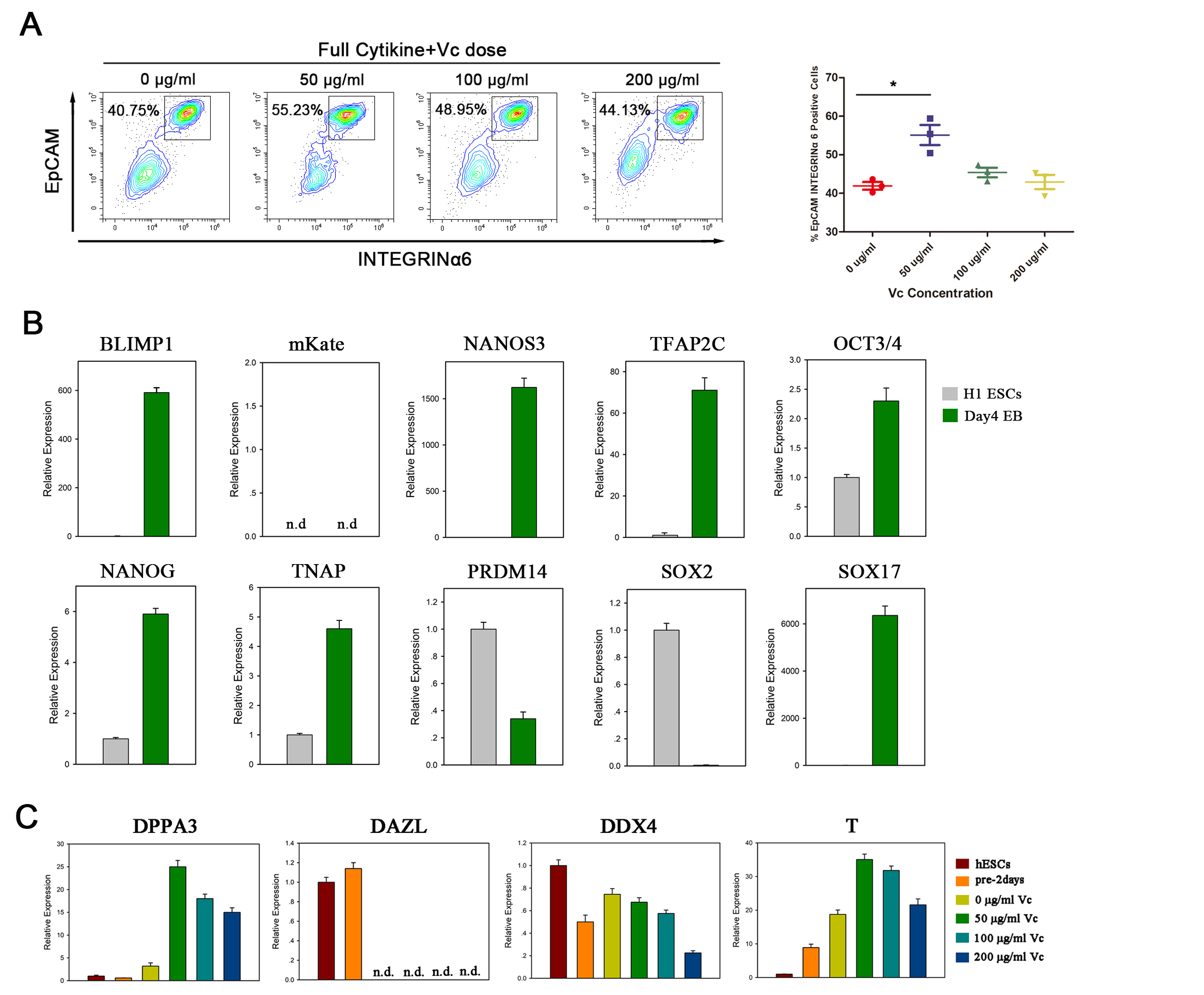

Supplement: Supplementary file 3 — Additional file 3: Figure S2. (A) FACS analysis of non-knockin H1 ESCs with EpCAM and INTEGRINα6 antibodies at day4; n=3 independent experiments; Data are presented as mean ± SD; Statistical analysis was performed by one-way analysis of variance. *p <0.05. (B) Expression analysis by RT-qPCR for day 4 EBs differentiated form native hESCs; Relative expression levels are shown with normalization to hESCs or day 4 EBs. n=3 independent experiments; Data are presented as mean ± SD. (C) Expression analysis of late PGC genes by RT-qPCR for day 4 EBs stimulated by different concentrations of Vitamin C (0, 50, 100, 200μg/ml). Relative expression levels are shown with normalization to hESCs. Error bars indicate mean ± SD from three independent biological replicates. n.d., not detected. [file 13287_2019_1427_MOESM3_ESM.tif]

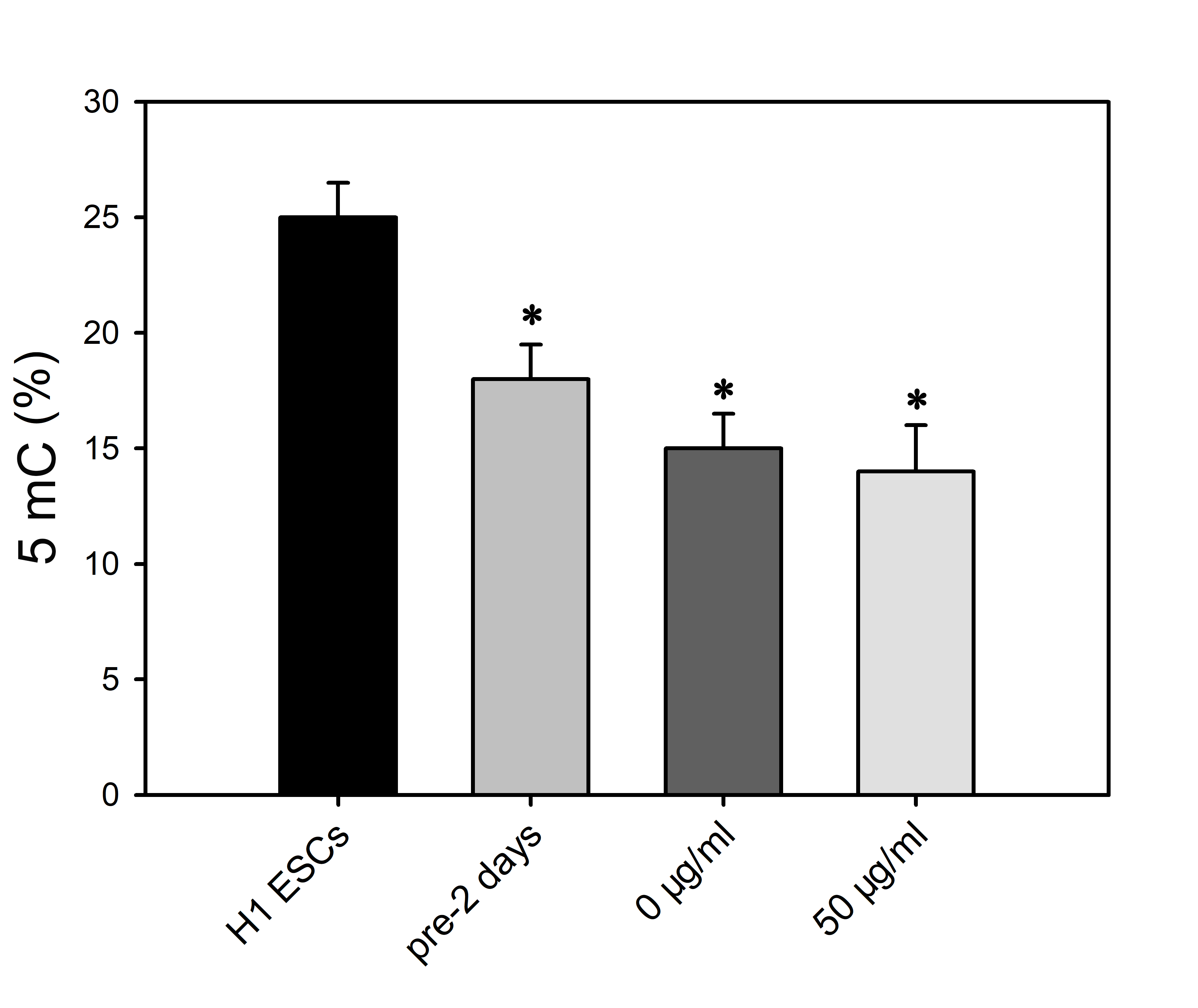

Supplement: Supplementary file 4 — Additional file 4: Figure S3. Analysis of 5mC levels by ELISA. n=3 independent experiments; Data are presented as mean ± SD; Statistical analysis was performed by one-way analysis of variance. *p <0.05. [file 13287_2019_1427_MOESM4_ESM.tif]
